# Computationally optimized molecularly imprinted electrochemical sensor based on biomass-derived biochar for paclobutrazol analysis in Radix Angelicae Sinensis

**DOI:** 10.1039/d6ra02926d

**Published:** 2026-07-03

**Authors:** Xin Wang, Xuxia Liu, Pen Jin, Delai Zhou, Guodi Lu, Jia Hou, Shijun Shao, Jian Xu, Fude Yang

**Affiliations:** a College of Pharmacy, Gansu University of Traditional Chinese Medicine Lanzhou 730000 China gszyyfd@163.com; b College of Chemical Engineering, Northwest Minzu University Lanzhou 730000 China; c Research Center for Natural Medicine and Chemical Metrology, Lanzhou Institute of Chemical Physics, Chinese Academy of Sciences Lanzhou 730000 China

## Abstract

Paclobutrazol (PBZ) is widely used in agriculture, but its residues in medicinal herbs may compromise product safety and quality. In this work, a molecularly imprinted electrochemical sensor was developed for the determination of PBZ in Radix Angelicae Sinensis. The sensor integrates Angelica stalk-derived biochar as a sustainable porous carbon substrate with a molecularly imprinted layer rationally designed through a combined computational approach. Density functional theory (DFT) calculations (Dmol3 module) were employed to screen the optimal functional monomer (*o*-phenylenediamine), and Forcite molecular dynamics simulations were further applied to determine the ideal template-to-monomer ratio, ensuring high-affinity recognition cavity formation. A deep eutectic solvent was introduced as a green eluent for template removal. Under optimized conditions, the MIP/ASB/GCE sensor exhibited a linear response from 50 to 450 nM, with an LOD of 11.49 nM and an LOQ of 38.30 nM. The sensor showed acceptable selectivity, reproducibility, repeatability, and storage stability.Recovery tests in spiked Radix Angelicae Sinensis samples gave recoveries of 105.80–109.30%. These results indicate that the proposed sensor is applicable for PBZ monitoring in complex herbal matrices and that the integration of biomass-derived carbon, molecular simulation-assisted imprinting, and green elution chemistry provides a useful strategy for MIP-based electrochemical sensing.

## Abstract 2

Angelica-stalk biochar, computation-guided molecular imprinting, and DES-assisted elution were integrated to enable selective electrochemical detection of paclobutrazol in Radix Angelicae Sinensis.

## Graphical abstract

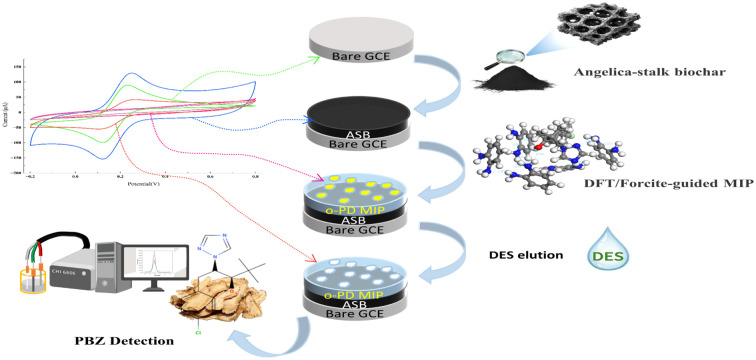

## 1 Introduction

Traditional Chinese medicine (TCM) has moved from experience-based practice toward an evidence-driven, industrialized and globally connected healthcare sector, where quality consistency, safety, and traceability are increasingly critical.^1,2^ Radix Angelica Sinensis (Danggui), approved by China's National Health Commission as a medicinal herb with dual food and medicinal properties, is renowned as the staple ingredient in nine out of ten traditional formulas.^3^ It has been utilised in traditional medicine for an extended period.^4^ However, as the cultivation of Danggui expands, pesticide residue concerns have emerged as a significant issue impacting its quality and safety.^5^

Paclobutrazol (PBZ), a widely employed plant growth regulator (PGR), is frequently utilized in the development of rhizome-based Chinese medicinal herbs. It significantly improves root and stem growth, resulting in robust roots and an increased number of roots.^6^ Its persistence in the environment, with a reported half-life ranging from 43 to 618 days, has raised concerns about the potential health risks associated with its residues in herbal products.^7^ In addition, previous studies have suggested that PBZ exposure may cause developmental toxicity, while residue application may also alter the accumulation of secondary metabolites in medicinal plants.^8,9^ Therefore, monitoring PBZ residues in Radix Angelicae Sinensis is important not only for contaminant control, but also for quality assurance of edible and medicinal herbal materials.

Chromatography-mass spectrometry provides excellent sensitivity and confirmatory capabilities, and is extensively utilised for the detection of PBZ residues in water, soil, and food;^8,10–12^ nevertheless, it generally necessitates complicated sample preparation and costly large-scale instrumentation. In contrast, immunoassays are more appropriate for rapid screening and point-of-care testing, although their accuracy and resistance to cross-interference are contingent upon the quality of the antibody.^13–15^ To address these challenges, electrochemical sensors, particularly molecularly imprinted electrochemical sensors (MIECS), have gained increasing attention because of their low cost, portability, rapid response, and suitability for real-sample analysis.^16–19^ The imprinted layer offers artificial receptor sites that are complementary to the template molecule in terms of size, shape, and functional groups, thereby enhancing selectivity, anti-interference capability, and applicability in complex real samples.^20–22^ Electropolymerized molecularly imprinted polymers are particularly noteworthy as the recognition layer can be directly formed on the electrode surface, with the film thickness and compactness adjustable *via* electrochemical parameters such as scan rate and cycle number.^23,24^

Numerous investigations demonstrate that elevated sensitivity is attained by integrating MIP films with sophisticated conductive materials, such Au nanoparticles, graphene derivatives, metal oxides, transition-metal chalcogenides, or conjugated polymer nanoparticles.^25–28^ These studies confirm that material engineering is an effective way to amplify MIP-based electrochemical signals, but they also increase fabrication complexity and introduce more variables that may affect reproducibility and scale-up.^20,29^ Compared with highly engineered multi-component nanocomposites, a single-source biomass carbon platform may simplify fabrication and improve consistency while still providing sufficient electrochemical performance for sensor construction.^25,30,31^ This is especially significant in molecularly imprinted electrochemical sensing, where the substrate must enable electron transfer while concurrently maintaining a stable and reproducible imprinting layer.

Another challenge in MIP development is the empirical nature of monomer selection and stoichiometry optimization. Since imprinting affinity and selectivity depend on template-monomer interactions, trial-and-error screening is inefficient and can reduce mechanistic clarity.^32^ Recent reviews highlight the increasing use of computational methods, such as density functional theory, molecular mechanics, and molecular docking, to rationalize monomer selection and optimize template-monomer interactions.^33,34^ This approach has significantly impacted electrochemical MIP development, with computationally guided strategies now supporting monomer choice, ratio optimization, and electropolymerization parameter tuning.^35–38^

In this work, we developed a novel molecularly imprinted electrochemical sensor based on Angelica sinensis stalk-derived biochar for the selective and sensitive determination of PBZ in Radix Angelicae Sinensis. The porous carbon substrate was prepared *via* phosphoric acid activation and high-temperature carbonization of discarded Angelica sinensis stalks, offering a cost-effective, eco-friendly, and highly conductive platform with abundant surface functional groups. To rationally design the MIP layer, DFT calculations using the Dmol3 module were employed to screen o-PD as the optimal functional monomer that forms the most stable pre-polymerization complex with PBZ. Forcite molecular dynamics simulations were further conducted to determine the optimal template-to-monomer ratio, ensuring the formation of high-affinity recognition cavities. A deep eutectic solvent (DES) was introduced as a green and efficient eluent for template removal, replacing conventional toxic organic solvents. The proposed sensor was systematically characterized, and its analytical performance was evaluated. Its practical applicability was successfully validated by detecting PBZ in spiked Radix Angelicae Sinensis samples. This work not only provides a reliable analytical tool for monitoring PBZ residues in complex herbal matrices but also establishes a comprehensive computational–experimental strategy for the rational design of high-performance MIP-based sensors.

## 2 Materials and methods

### 2.1 Chemicals and materials

Angelica Sinensis stalks were collected from the Shouyang Medicinal Herb Garden herbal cultivation base in Longxi County, Gansu Province. Radix Angelica Sinensis was provided by the Affiliated Hospital of Gansu University of Chinese Medicine. Phosphoric acid (H_3_PO_4_, 85%),sodium hydroxide (NaOH, 99%)were bought from Shanghai Macklin Biochemical Co., Ltd. Potassium ferrocyanide (K_4_[Fe(CN)_6_],99%), potassium ferricyanide (K_3_[Fe(CN)_6_],99%), Paclobutrazol (99%), ferulic acid(99.5%), triazophos(97%), glyphosate(99.5%), *o*-phenylenediamine (o-PD, 99.5%),ethylene glycol (EG, 98%), choline chloride (ChCl, 99%), disodium hydrogen phosphate (Na_2_HPO_4_,98%), sodium dihydrogen phosphate (NaH_2_PO_4_,98%), and other reagents were analytical grade and bought from Shanghai Aladdin Biochemical Technology Co., Ltd. The DES used as the eluent was prepared using the method described in our previous report.^38^ Phosphate buffer solutions (0.1 M) were prepared from 0.1 M Na_2_HPO_4_ and NaH_2_PO_4_, and the desired pH values were obtained by adjustment with 0.1 M HCl or 0.1 M NaOH.^39^ Ferro-/ferricyanide redox couple solution was prepared from a mixture of 5 mM K_3_[Fe(CN)_6_]/K_4_[Fe(CN)_6_] (1 : 1 M ratio) in 0.1 M KCl.

### 2.2 Apparatus

The surface morphology and elemental distribution of ASB were analyzed by scanning electron microscopy (SEM, ZEISS Sigma 300, Germany) equipped with an energy-dispersive spectroscopy system (EDS, OXFORD Xplore). Fourier-transform infrared spectroscopy (FTIR, Thermo Nicolet 380, USA) and X-ray photoelectron spectroscopy (XPS, Thermo Scientific K-Alpha, USA) were employed to investigate the surface functional groups, chemical bonding, and valence states of the elements. The pore characteristics of ASB were evaluated from N_2_ adsorption–desorption isotherms at 77 K using an automated sorption analyzer (Micromeritics ASAP 2460, USA). Raman spectroscopy (Horiba LabRAM HR Evolution, Japan) was used to probe its structural features. Electrochemical measurements, including cyclic voltammetry (CV), differential pulse voltammetry (DPV), and electrochemical impedance spectroscopy (EIS), were carried out on a CHI660E electrochemical workstation(Austin, TX, USA) with a conventional three-electrode configuration, consisting of the modified electrode as the working electrode, a Hg/Hg_2_Cl_2_ electrode as the reference electrode, and a platinum wire as the counter electrode.

### 2.3 Computersimulation

Material Studio 2023 was used to investigate the molecular imprinting system at the atomic level. The interactions between PBZ and different candidate functional monomers were first evaluated by DMol3 calculations using the GGA-PBE functional with the DNP(3.5) basis set and the COSMO solvent model for water.^40^ Geometry optimization was performed with convergence criteria of 1.0 × 10^−6^ Ha per atom for SCF tolerance, 1.0 × 10^−5^ Ha per atom for energy, 0.002 Ha Å^−1^ for maximum force, and 0.005 Å for maximum displacement.^38^ Based on the calculated interaction energies, o-PD was selected as the functional monomer.

The pre-polymerization systems with different PBZ/o-PD molar ratios (1 : 2, 1 : 3, 1 : 4, 1 : 5 and 1 : 6) were constructed in the Amorphous Cell module and optimized using the Forcite module with the COMPASS III force field. Water molecules were introduced into each simulation box to mimic the solution environment. After structural relaxation and energy minimization, the interaction energy between PBZ and o-PD in the solvated system was estimated according to:Δ*E*_int_ = *E*_PBZ+o-PD+H_2_O_ − *E*_o-PD+H_2_O_ − *E*_PBZ+H_2_O_ + *E*_H_2_O_where a more negative Δ*E*_int_ indicates a stronger and more stable interaction between PBZ and o-PD under the same simulation conditions. Among the tested molar ratios, the 1 : 5 PBZ/o-PD system exhibited the lowest interaction energy, suggesting the most favorable pre-polymerization configuration and therefore being selected as the optimal stoichiometric ratio.

It is important to note that the above DMol3 and Forcite calculations were performed under a neutral aqueous model, whereas the experimental electropolymerization was carried out in PBS at pH 4.5. Therefore, the possible influence of pH on the speciation of the interacting molecules should be considered when interpreting the calculated interaction patterns.

Available regulatory data indicate that paclobutrazol does not dissociate within the environmentally relevant pH range and remains stable to hydrolysis at pH 4–9, suggesting that PBZ is predominantly present in its neutral molecular form under the experimental condition of pH 4.5.^41^ In contrast, o-PD has reported p*K*_a_ values of <2 and 4.47, indicating that neutral and monoprotonated species may coexist around pH 4.5.^42^ Therefore, an additional PBZ-o-PDH^+^ model was constructed and optimized to evaluate the effect of monoprotonation on the template-monomer interaction.

The calculated interaction energy of the PBZ-o-PDH^+^ complex was −8.979 kcal mol^−1^, which was more negative than that of the neutral PBZ-o-PD complex. This result indicates that the monoprotonated o-PD species can form a more stable pre-polymerization complex with PBZ under mildly acidic conditions. The enhanced interaction may be attributed to the strengthened hydrogen-bonding and electrostatic interactions between PBZ and o-PDH^+^. Therefore, although the neutral-state calculations were used as the initial comparative screening model, the additional protonation-state calculation further supports the selection of o-PD as the functional monomer under the experimental pH condition.

### 2.4 Synthesis of ASB

ASB was prepared by a phosphoric-acid activation and carbonization method.^43^ Briefly, Angelica stalks were thoroughly rinsed with distilled water, air-dried for 2 days, and pulverized to pass through a 150 mesh sieve. Then, 2 g of the obtained powder was immersed in H_3_PO_4_ solutions of different concentrations (3.6, 4.6, and 5.6 M) for 24 h. After impregnation, the samples were dried at 80 °C for 12 h and subsequently carbonized in a tubular furnace at 500 °C for 2 h under a nitrogen atmosphere with a heating rate of 5 °C min^−1^. The carbonized products were washed with 5% HCl, followed by repeated rinsing with distilled water until neutral pH, and then dried again at 80 °C for 12 h. The obtained biochar samples were denoted according to the concentration of the activating solution. Among them, the sample prepared with 4.6 M H_3_PO_4_ showed the most favorable properties and was therefore used in the following experiments.

### 2.5 Construction of ASB/GCE

To overcome the limited active surface and lack of molecular recognition ability of bare GCE,^18^ ASB was introduced as a conductive modifier before MIP construction. The bare glassy carbon electrode (GCE, 3 mm in diameter) was pretreated according to a previously reported procedure, including mechanical polishing, ultrasonic cleaning, and electrochemical activation.^39^ Then, 3 mg of the as-prepared ASB was dispersed in 1 mL of deionized water, followed by the addition of 15 µL of Nafion solution. The mixture was ultrasonicated to obtain a uniform suspension. Subsequently, 7 µL of the resulting suspension was drop-cast onto the GCE surface and dried at room temperature. In this process, Nafion served as a film-forming binder, helping to immobilize the ASB layer on the electrode surface and improve the stability of the modified interface.^44,45^

### 2.6 Construction of MIP/ASB/GCE

**Scheme 1:**
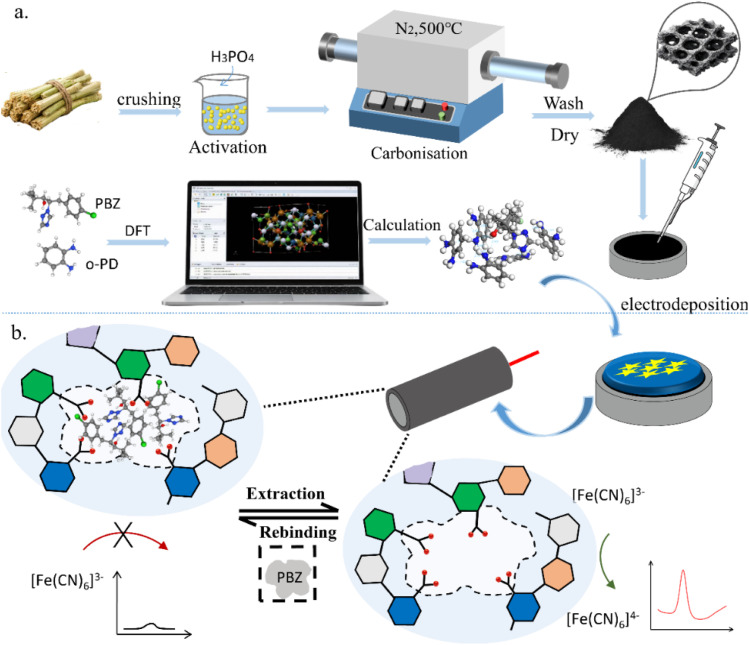
Schematic illustration of (a) the fabrication process of the MIP/ASB/GCE and (b) the recognition mechanism for the selective determination of PBZ.

The MIP layer was fabricated on the ASB-modified GCE by electropolymerization in 0.1 M PBS (pH 4.5) containing 1.0 mM PBZ and 5.0 mM o-PD (Scheme 1). The electropolymerization process was carried out by CV for 10 cycles at 50 mV s^−1^ within a potential window of −0.2 to 0.8 V. The resulting electrode was then eluted with DES for 5 min to remove the template and obtain the MIP/ASB/GCE. A corresponding NIP/ASB/GCE was prepared in parallel using the same procedure without adding PBZ.

To evaluate the elution efficiency of the green DES system, several conventional and DES-based eluents were comparatively investigated under the same elution time (5 min). The tested eluents included methanol/acetic acid (9 : 1, v/v), 0.1 M NaOH/acetonitrile (1 : 9, v/v), 0.1 M NaOH/methanol (1 : 9, v/v), and ChCl/EG DES with molar ratios of 1 : 2, 1 : 3, and 1 : 4. The elution performance was assessed by the recovery of the probe current after template removal and the subsequent rebinding response toward PBZ under the same conditions.

### 2.7 Electrochemical measurements

Prior to electrochemical testing, the modified electrodes were immersed in PBS containing the desired PBZ concentration for 7 minutes, followed by gentle rinsing with ultrapure water to remove excess or loosely bound PBZ. All electrochemical measurements were performed in 0.1 M KCl containing 5.0 mM Fe(CN)_6_^3−/4−^ as the redox probe. CV was carried out over a potential range from −0.2 to 0.8 V at a scan rate of 100 mV s^−1^. EIS was measured over the frequency range of 100 kHz to 1 Hz, with an initial potential of 0.192 V. For DPV, measurements were taken from −0.2 to 0.8 V, with a step potential of 4 mV, pulse width of 0.05 s, pulse amplitude of 0.05 V, and pulse period of 0.5 s.^46,47^

### 2.8 Preparation of real samples

The Angelica sinensis sample was ground into powder and passed through a 100-mesh sieve. A 1.0 g portion of the powder was extracted with 20 mL of ethanol/water (8 : 2, v/v). The extraction was carried out by ultrasonication at 60 °C for 30 min, with the mixture shaken every 5 min to maintain uniform dispersion. After centrifugation at 8000 rpm for 20 min, the supernatant was collected and concentrated to approximately 1–2 mL using a rotary evaporator. The resulting extract was then diluted to 10 mL with PBS (pH 7.0), filtered through a 0.22 µm membrane, and stored at 4 °C for subsequent analysis.

## 3 Results and discussion

### 3.1 Computational simulation for monomer screening and stoichiometric optimization

**Fig. 1:**
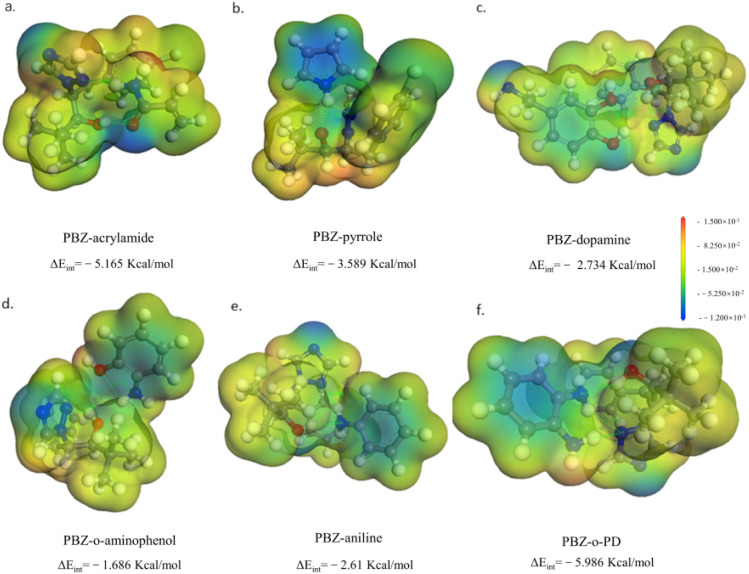
DFT-calculated complexes between PBZ and different candidate functional monomers used for monomer screening: (a) acrylamide, (b) pyrrole, (c) dopamine, (d) *o*-aminophenol, (e) aniline, and (f) *o*-phenylenediamine (o-PD). The electrostatic potential surface distributions and corresponding interaction energies (Δ*E*_int_) are presented for comparison.

To rationally design the PBZ-imprinted electrochemical interface, a two-step computational strategy was adopted. First, the interactions between PBZ and six candidate functional monomers were evaluated using the DMol3 module in Material Studio. As shown in Fig. 1, among the tested monomers, o-PD showed the most favorable interaction with PBZ, while acrylamide ranked second and the other monomers exhibited distinctly weaker affinities. In general, a more negative interaction energy indicates a stronger non-covalent interaction and a more stable pre-polymerization complex, which is favorable for the formation of well-defined imprinted recognition sites.^35,48^ Further inspection of the optimized structures revealed that the PBZ-o-PD complex was stabilized by three hydrogen bonds with bond lengths of 1.942, 2.246, and 2.412 Å, whereas the PBZ–acrylamide complex contained only two hydrogen bonds with bond lengths of 1.889 and 1.865 Å. Although the individual hydrogen bonds in the PBZ–acrylamide complex were slightly shorter, its overall interaction energy was still less favorable than that of PBZ-o-PD. This suggests that the stability of the template-monomer assembly is not governed solely by the strength of isolated hydrogen bonds, but rather by the combined contribution of hydrogen-bond number, geometric complementarity, and overall configurational cooperativity.^37,49^ Considering both the calculated interaction energies and the known electropolymerization suitability of o-PD, o-PD was selected as the functional monomer for subsequent imprinting design.

Because the experimental electropolymerization was carried out in PBS at pH 4.5, the protonation state of o-PD was further considered. PBZ is expected to remain predominantly neutral under this condition, whereas o-PD may coexist as neutral and monoprotonated species. Therefore, PBZ-o-PDH^+^ model was constructed(Fig. S1). The PBZ-o-PDH^+^ complex exhibited a more negative interaction energy of −8.979 kcal mol^−1^ than the neutral PBZ-o-PD complex, indicating that partial protonation of o-PD under mildly acidic conditions can further strengthen the template-monomer interaction through enhanced hydrogen-bonding and electrostatic contributions. These results support the selection of o-PD from two aspects: the neutral-state model provides a unified baseline for comparing monomer affinity, while the monoprotonated model offers a more experimentally relevant explanation for the favorable PBZ-o-PD interaction at pH 4.5. Therefore, the computational results should be interpreted as qualitative or semi-quantitative descriptors for rational monomer screening, with the final preparation conditions determined by subsequent experimental optimization.

**Fig. 2:**
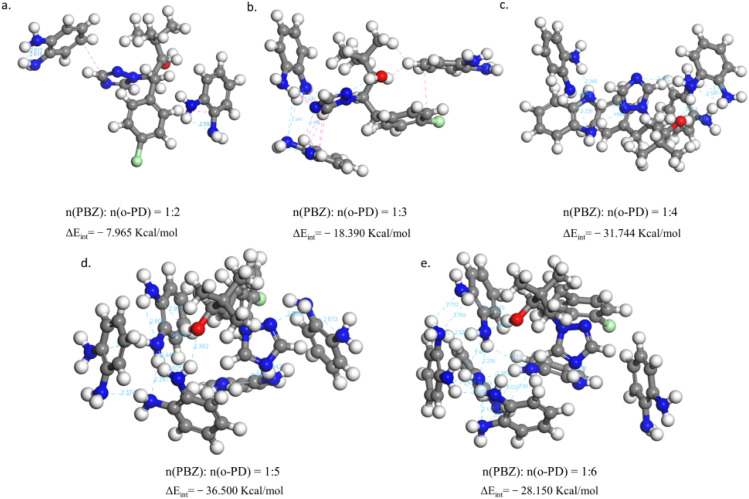
Forcite-optimized configurations and interaction energies of PBZ/o-PD pre-polymerization assemblies at different molar ratios: (a) 1 : 2, (b) 1 : 3, (c) 1 : 4, (d) 1 : 5, and (e) 1 : 6. The corresponding interaction energies (Δ*E*_int_) are shown below each optimized structure.

After monomer screening, the stoichiometric effect of the PBZ/o-PD pre-polymerization system was further investigated using the Forcite module. Fig. 2 illustrates that as the quantity of o-PD molecules increased from 2 to 5, the interaction energy became increasingly negative, signifying that the addition of monomer molecules enhanced the cooperative interaction network surrounding PBZ and augmented the stability of the pre-polymerization assembly. This trend is consistent with previous computationally guided MIP studies showing that the template-to-monomer ratio strongly affects both the strength of template-monomer interactions and the quality of the recognition cavities eventually formed in the imprinted polymer. At the 1 : 5 ratio, PBZ was surrounded by a more complete and balanced interaction environment, suggesting that multiple o-PD molecules could stabilize the template from different directions and thereby maximize the overall assembly stability. Such a multi-point cooperative arrangement is expected to favor the generation of more complementary imprinting cavities during the subsequent electropolymerization process. Interestingly, when the ratio was further increased to 1 : 6, the interaction energy became less favorable, increasing from −36.500 to −28.150 kcal mol^−1^. This result suggests that excessive monomer addition does not continuously improve template-monomer interaction; instead, an overabundance of o-PD molecules may introduce steric crowding and configurational competition, thereby weakening the overall stability of the pre-polymerization system. Similar studies have emphasized that the optimal monomer/template ratio is not simply the highest monomer content, but rather the ratio at which strong intermolecular interaction and favorable cavity formation are simultaneously achieved.^50,51^

### 3.2 Materials characterization

**Fig. 3:**
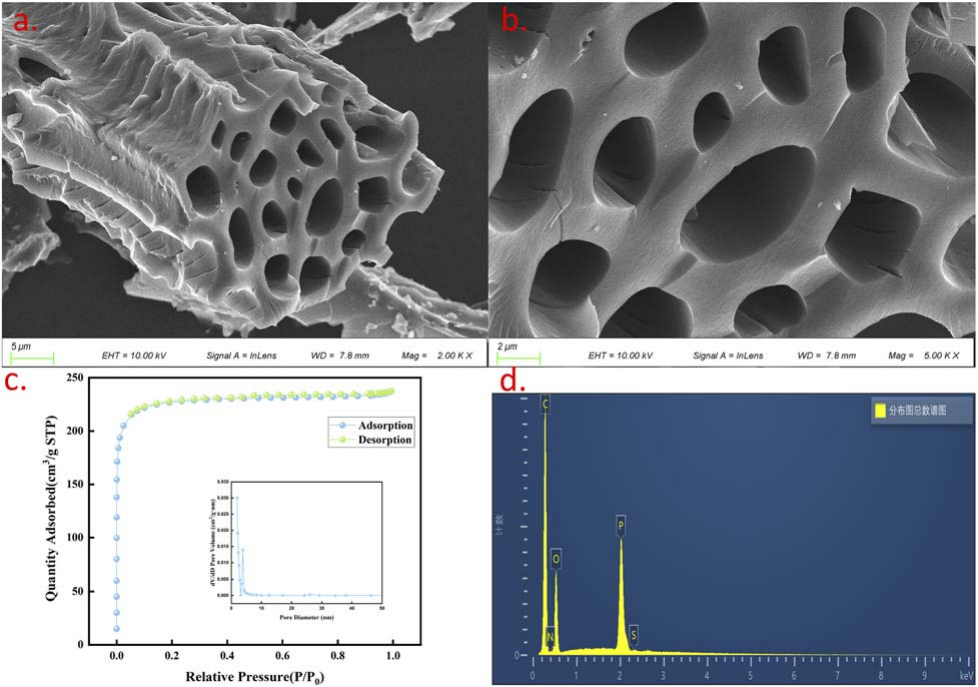
Morphological, textural and elemental characterization of ASB. (a and b) SEM images of ASB at different magnifications. (c) N_2_ adsorption–desorption isotherms with the inset showing the pore size distribution. (d) EDS spectrum of ASB.

Fig. 3a and b illustrate that the SEM reveals ASB maintains a continuous three-dimensional carbon structure characterized by numerous open, interconnecting macropore-sized channels. This morphology is significant for the present sensing platform because the open framework can facilitate electrolyte infiltration and analyte transport, while the continuous carbon walls provide a stable substrate for the subsequent electropolymerized MIP layer. In biomass-derived electrochemical materials, such an accessible macroporous scaffold is generally considered beneficial because it improves interfacial accessibility without sacrificing structural integrity. The gas sorption data(Fig. 3c) further clarify where the effective interface originates. The N_2_ adsorption–desorption curve shows a sharp uptake at low relative pressure followed by an early plateau, indicating that the surface area is dominated by small pores. This interpretation is consistent with the parameters (Table S1). Therefore, most of the accessible surface area is contributed by microporous domains embedded within the carbon walls, whereas the larger openings observed by SEM mainly serve as transport channels. This hierarchical organization is particularly advantageous for sensing, because it combines high interfacial site density with efficient mass transfer of electrolyte ions, redox probes, and analyte molecules.^25^

**Fig. 4:**
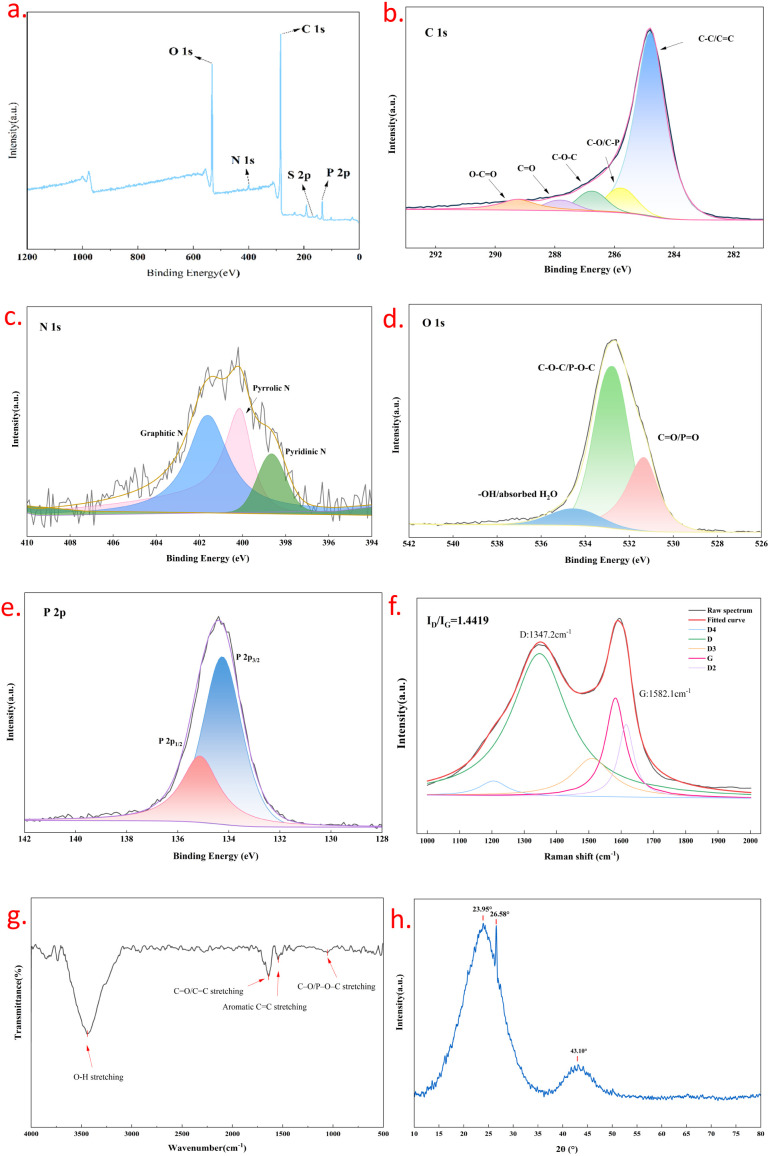
Surface chemical and carbon-structure characterization of ASB. (a) XPS survey spectrum; (b) C 1s spectrum; (c) N 1s spectrum; (d) O 1s spectrum; (e) P 2p spectrum; (f) Raman spectrum with Lorentzian deconvolution in the range of 1000–2000 cm^−1^; (g) FTIR spectrum and (h) XRD pattern.

EDS (Table S2, Fig. 3d and S2) and XPS (Fig. 4a) collectively indicate that ASB is a phosphorus-rich, oxygen-functionalized carbon rather than an inert biochar. The elevated phosphorus content indicates that H_3_PO_4_ activation not only creates porosity but also leaves phosphorus-containing surface species in the carbon matrix. These groups can increase surface polarity and wettability, improve electrolyte accessibility, and promote interfacial electrochemical activity, which is expected to be beneficial for subsequent functional coating.^52,53^ The high-resolution C 1s spectrum (Fig. 4b) was fitted into C–C/C=C, C–O/C–P, C–O–C, C=O, and O–C=O components. The dominant C–C/C=C component confirms the formation of a conjugated carbon backbone, which provides conductive pathways for electron transfer. Meanwhile, the oxygen- and phosphorus-containing carbon species indicate abundant polar functional groups on the ASB surface. These groups can enhance the dispersion and wettability of ASB and provide anchoring sites for the subsequent electropolymerization of *o*-phenylenediamine, thereby facilitating stable construction of the imprinted polymer layer. The N 1s spectrum (Fig. 4c) shows pyridinic N, pyrrolic N, and graphitic N components. Pyridinic and pyrrolic N can introduce defect sites and improve the surface affinity of the carbon material, while graphitic N is favorable for modulating the electronic structure and enhancing charge-transfer capability. The O 1s spectrum (Fig. 4d) further reveals C–O–C/P–O–C, C=O/P=O, and –OH/adsorbed H_2_O species, confirming the presence of oxygen- and phosphorus-related functional groups. In addition, the P 2p spectrum (Fig. 4e) shows the characteristic P 2p_3/2_ and P 2p_1/2_ doublet, further verifying the introduction of phosphorus-containing species during phosphoric-acid activation. These heteroatom-containing groups contribute to the hydrophilic and chemically active surface of ASB, which is important for improving the adhesion and stability of the MIP film.

The FTIR spectrum (Fig. 4g) is consistent with the XPS results. The broad band around 3440 cm^−1^ is assigned to O–H stretching, while the bands around 1639 and 1544 cm^−1^ are related to C=O/C=C stretching and aromatic C=C skeletal vibration, respectively. The band near 1054 cm^−1^ can be attributed to C–O/P–O–C stretching vibrations. These results confirm that H_3_PO_4_ activation not only promotes the formation of aromatic carbon domains but also introduces oxygen- and phosphorus-containing functional groups, which are favorable for interfacial compatibility with the MIP layer. Raman spectroscopy was used to evaluate the disorder degree and graphitic domains of ASB. As shown in Fig. 4f, the Raman spectrum was deconvoluted using Lorentzian fitting in the range of 1000–2000 cm^−1^. The fitted D and G bands were located at approximately 1347.2 and 1582.1 cm^−1^, respectively. The D band is associated with structural defects and disordered carbon, whereas the G band corresponds to the in-plane vibration of sp^2^ carbon domains. The calculated *I*_D_/*I*_G_ value of 1.4419 indicates that ASB contains abundant defects while retaining partially graphitized sp^2^ carbon networks. Such structure is advantageous for electrochemical sensing, because the defect sites and heteroatom-associated carbon environments can provide electroactive sites, while the sp^2^ carbon framework supports electron-transfer pathways.^54,55^ The XRD pattern further confirms the disordered carbon structure of ASB (Fig. 4h). Two broad diffraction bands at approximately 23.95° and 43.10° can be assigned to the (002) and (100)/(101) planes of disordered carbon, respectively. The broad diffraction features indicate that ASB mainly possesses an amorphous/turbostratic carbon structure. The interlayer spacing calculated from the broad (002) band is 0.3712 nm, larger than that of ideal graphite, suggesting loosely stacked and defective carbon layers. A narrow diffraction peak at around 26.58° may arise from locally ordered graphitic domains or trace crystalline residues from the biomass precursor. Overall, these results demonstrate that ASB possesses a defect-rich, heteroatom-functionalized, and partially graphitized carbon structure, enabling it to serve as a conductive and chemically active substrate for constructing the PBZ-imprinted electrochemical sensor.

### 3.3 Electrochemical characterization

**Fig. 5:**
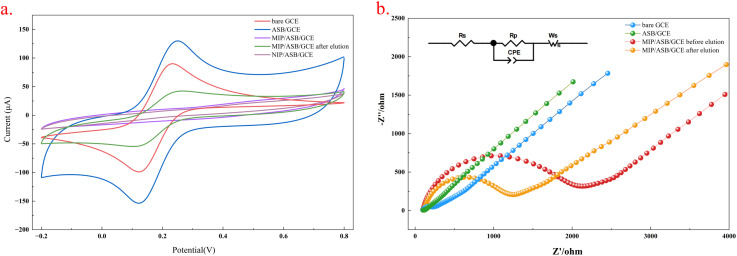
(a) CVs curves for stepwise modifications including bare GCE, ASB/GCE, MIP/ASB/GCE, MIP/ASB/GCE after elution, and NIP/ASB/GCE. (b) EIS characterization of electrode modification. Nyquist plots of bare GCE, ASB/GCE, MIP/ASB/GCE before and after elution, the inset shows the equivalent circuit used for fitting.

The sequential electrochemical behavior of the modified electrodes was examined using CV and EIS in a solution of 5.0 mM Fe(CN)_6_^3−/4−^ with 0.1 M KCl. As shown in Fig. 5a, bare GCE exhibits a pair of well-defined redox peaks. After modification with ASB, the peak current increases markedly, indicating that ASB enhances interfacial electron transfer by providing a more accessible and electroactive surface. This is consistent with the porous and defect-rich nature of ASB. After electropolymerization, the current response of MIP/ASB/GCE decreases significantly, indicating that the polymer film acts as a compact barrier to probe diffusion and electron transfer. A similarly low response is observed for NIP/ASB/GCE, confirming that the polymer matrix itself is responsible for the blocking effect. After template elution, however, the current of MIP/ASB/GCE increases again, showing that removal of PBZ generates accessible imprinted cavities and partially restores probe transport. This current recovery is characteristic of successful imprinting rather than simple film loss.

The EIS results in Fig. 5b support the same conclusion. To provide quantitative evidence for the interfacial changes, the impedance spectra were further fitted using the equivalent circuit shown in Fig. 5b, and the corresponding parameters are summarized in Table S3. In the selected circuit, *R*_s_ represents the solution resistance, *R*_p_ corresponds to the interfacial charge-transfer resistance, CPE denotes the constant phase element, and *W*_s_ represents the finite-length Warburg diffusion contribution. Similar circuit-based analyses have been widely used in EIS studies of electrochemical sensors.^56,57^ The fitted results showed that ASB/GCE exhibited a much lower *R*_p_ value than bare GCE, indicating improved interfacial electron transfer after ASB modification. After formation of the imprinted polymer film, the *R*_p_ value increased markedly, reflecting the blocking effect of the polymer layer toward the redox probe. Following template removal, *R*_p_ decreased again, suggesting the formation of accessible imprinted cavities and partial recovery of the probe-transfer pathway. The much less pronounced semicircle of ASB/GCE is also consistent with its low fitted *R*_p_ value and more diffusion-dominated response.

The electroactive surface area was further estimated from scan-rate-dependent CVs using the Randles–Sevcik equation,*I*_p_ = (2.69 × 10^5^)*n*^3/2^*ν*^1/2^*A*_eff_*D*^1/2^*C*_0_

Under semi-infinite diffusion control, the peak current is proportional to the square root of the scan rate. As shown in Fig. S3, both electrodes exhibit a good linear relationship between anodic peak current and *ν*^1/2^, indicating a diffusion-controlled process.^58,59^ The larger slope of ASB/GCE confirms that ASB effectively enlarges the electroactive interface, providing more accessible sites for probe exchange and subsequent MIP deposition. The calculated *A*_eff_ values for the bare GCE and ASB/GCE are 0.0885 cm^2^ and 0.1531 cm^2^, respectively. The larger electroactive surface area of ASB/GCE confirms that ASB enlarged the accessible electrochemical interface, providing more active sites for probe exchange and subsequent MIP deposition. Together with the lower fitted *R*_p_ value, these results indicate that ASB improves both the available electroactive area and the interfacial electron-transfer efficiency, thereby supporting its use as a conductive substrate for constructing the PBZ-imprinted sensing interface.

### 3.4 Optimization conditions

To achieve optimal sensing performance, the key fabrication and detection parameters were systematically optimized before analytical evaluation. In this work, the optimization was performed based on the DPV-derived current difference Δ*I*, because DPV was used as the analytical method for PBZ detection with Fe(CN)_6_^3−/4−^ as the redox probe. Similar Δ*I*-based optimization strategies have been widely used in probe-mediated MIP electrochemical sensors.^36,60^ Δ*I* was calculated as the difference between the peak current after template removal and that after PBZ rebinding, expressed as Δ*I* = *I*_elution_ – *I*_rebinding_. A larger Δ*I* indicates stronger suppression of redox-probe transport caused by PBZ rebinding in the imprinted cavities, thereby reflecting a higher sensing response. Although CV was employed for the electropolymerization of the MIP film, the CV profiles mainly reflect the polymer growth process, whereas the DPV-derived Δ*I* directly represents the final sensing performance after template removal and target rebinding. Therefore, the coating volume of biochar, electropolymerization pH, scan rate, incubation time and eluent composition were optimized according to the maximum Δ*I* value while keeping the other parameters constant.

**Fig. 6:**
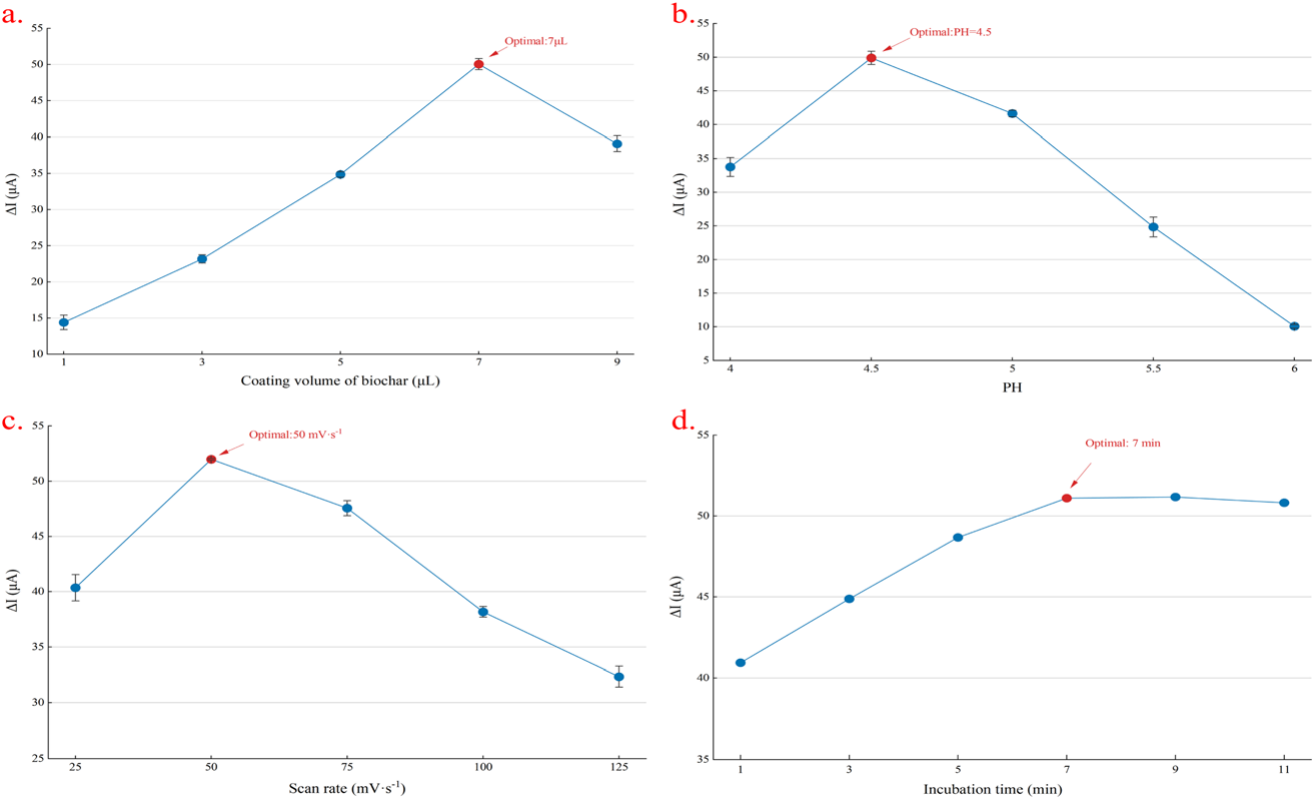
DPV-response-based optimization of experimental conditions: (a) coating volume of biochar, (b) pH of the electropolymerization solution, (c)scan rate during electropolymerization and (d) incubation time.

The quantity of ASB deposited on the electrode surface significantly affected the sensing response. As illustrated in Fig. 6a, Δ*I* increased with increasing coating volume and reached a maximum at 7 µL, then decreased at higher loading. This trend indicates that a moderate ASB layer is required to fully exploit the high-area and conductive characteristics of the biochar substrate. Insufficient loading cannot provide enough interfacial amplification, whereas excessive loading likely forms a thicker coating that increases the diffusion path of the redox probe and partially weakens charge transfer. Therefore, 7 µL was selected as the optimal coating volume.

The pH of the electropolymerization medium significantly affected the final sensing response of the MIP sensor. MIP films were prepared by CV electropolymerization at pH from 4.0 to 6.0. As shown in Fig. 6b, the highest Δ*I* was obtained at pH 4.5, indicating that a mildly acidic medium was optimal for PBZ imprinting. The superior response at pH 4.5 can be attributed to the balance between PBZ-o-PD pre-assembly and CV electropolymerization of o-PD. Under this condition, PBZ is expected to remain mainly neutral, while o-PD may coexist as neutral and monoprotonated species. The Fig. S3 showed that PBZ-o-PDH^+^ had a more negative interaction energy than neutral PBZ-o-PD, suggesting that partial protonation of o-PD can strengthen the template-monomer interaction. At pH 4.0, excessive protonation of o-PD may disturb the interaction geometry and film growth, whereas at pH 5.0–6.0, the reduced fraction of monoprotonated o-PD and altered electropolymerization behavior may be less favorable for producing accessible recognition cavities. Therefore, pH 4.5 was chosen for subsequent sensor fabrication.

The scan rate during electropolymerization significantly influenced the quality of the imprinted film. As illustrated in Fig. 6c, Δ*I* initially ascended and subsequently descended with a rising scan rate, attaining a peak at 50 mV s^−1^. A reduced scan rate results in an extended residence time within the oxidation region, facilitating more extensive electropolymerization, perhaps resulting in an excessively thick or dense coating that obstructs cavity accessibility. Conversely, at an elevated scan rate, the polymer deposition may occur too swiftly to yield a sufficiently homogeneous and ordered imprinted layer. Consequently, 50 mV s^−1^ was designated as the best scan rate, as it offers an appropriate equilibrium between film production and cavity accessibility.

As depicted in Fig. 6d, the response increased rapidly with incubation duration, thereafter leveling out and attaining a stable maximum at 7 minutes. This trend suggests that PBZ rebinding to the imprinted cavities occurs rapidly, allowing adsorption equilibrium to be attained in a little period. Prolonging the incubation period did not yield a significant increase in signal, suggesting that the majority of available recognition sites were already filled. Consequently, 7 minutes was designated as the best incubation duration.

**Fig. 7:**
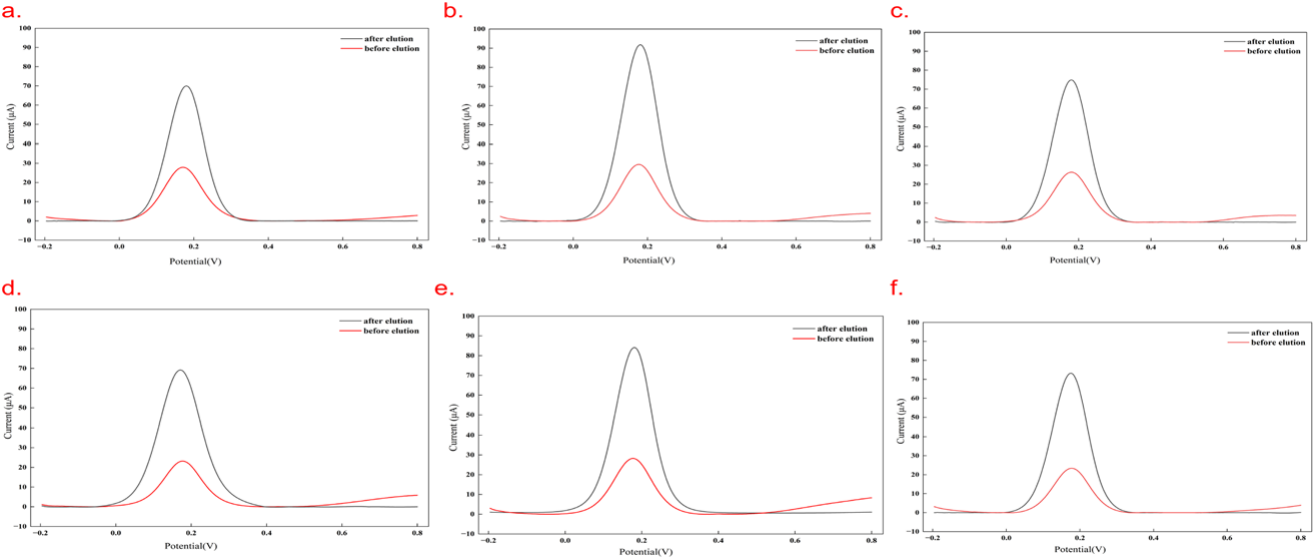
DPV responses of the MIP sensor before and after elution using different eluents: (a) CH_3_OH/CH_3_COOH = 9 : 1, (b) CH_3_OH/0.1 M NaOH = 9 : 1, (c) CH_3_CN/0.1 M NaOH = 9 : 1, (d) ChCl/EG = 1 : 2, (e) ChCl/EG = 1 : 3, and (f) ChCl/EG = 1 : 4.

The elution step was evaluated not only by the current recovery after template removal but also by the rebinding-derived current difference. As shown in Fig. 7a and S4, although CH_3_OH/CH_3_COOH = 9 : 1 caused a relatively large increase in DPV current after elution, its subsequent PBZ rebinding response was not optimal. This indicates that strong current recovery alone cannot be regarded as evidence of effective imprinting, because excessive swelling or disruption of the polymer layer may reopen probe pathways while partially weakening the recognition microenvironment.^61^ Similarly, alkaline eluents, including CH_3_OH/0.1 M NaOH and CH_3_CN/0.1 M NaOH (Fig. 7b and c), produced a more pronounced increase in the DPV current after elution but showed lower rebinding responses. This suggests that alkaline conditions may disturb the non-covalent interactions and local microenvironment of the o-PD-based imprinted layer, thereby reducing the accessibility or fidelity of the recognition sites. In contrast, ChCl/EG-based DESs (Fig. 7d–f) offered a milder and more selective elution environment through their hydrogen-bond donor/acceptor network and tunable polarity.^62^ The DES ratio was also important: ChCl/EG = 1 : 2 may suffer from high viscosity and limited mass transfer,^63^ whereas excessive EG may alter the hydrogen-bonding network and reduce extraction efficiency. ChCl/EG = 1 : 3 provided the highest Δ*I* after PBZ rebinding, suggesting the best balance between template removal, mass transfer, and preservation of accessible imprinted cavities. Thus, ChCl/EG = 1 : 3 was selected as the optimal green eluent.

### 3.5 Quantitative detection of PBZ

**Fig. 8:**
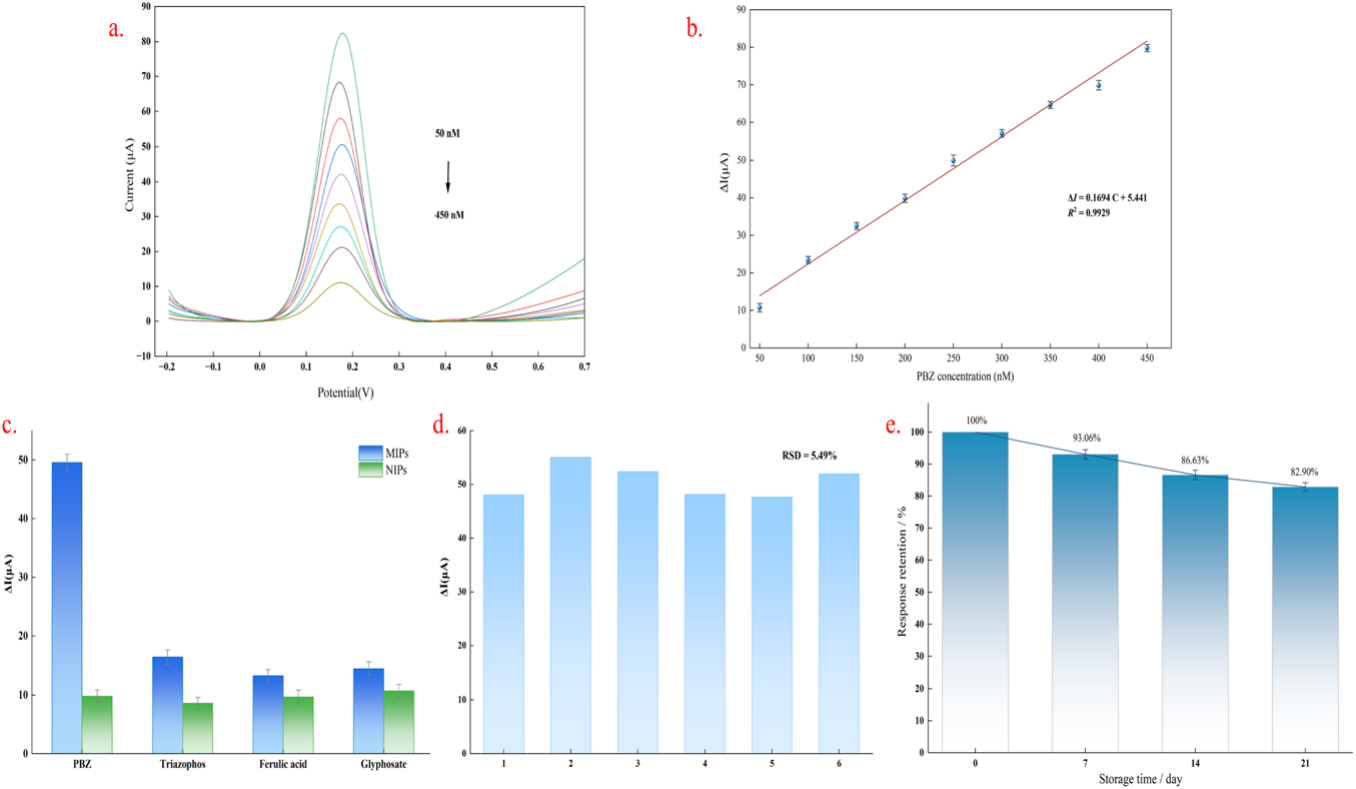
(a) DPV tests of different concentrations of PBZ; (b)Calibration curve for PBZ detection using the MIP-based sensor; (c) the current responses for PBZ and other compounds; (d)reproducibility of the fabricated sensor and (e) Long-term storage stability of the MIP-based sensor over 21 days.

The analytical performance of the proposed sensor was evaluated using PBZ standard solutions. Different concentrations of PBZ were prepared, and the MIP/ASB/GCE was incubated in these solutions under the optimized conditions. Subsequently, electrochemical measurements were indirectly carried out by DPV using the Fe(CN)_6_^3−/4−^ redox probe. The re-association of PBZ molecules within the imprinted cavities on the MIP/ASB/GCE surface impeded the transport of the redox probe, leading to a reduction in the DPV peak current (Fig. 8a).

As the PBZ concentration increased, the peak current gradually decreased, resulting in a progressive increase in the Δ*I*. Fig. 8b illustrates the correlation between Δ*I* and PBZ concentration. The proposed sensor demonstrated a linear response over the concentration range of 50–450 nM, adhering to the regression equation Δ*I* (µA) = 0.1694C (nM) + 5.441, with a correlation coefficient of 0.9929. The sensitivity, defined as the slope of the calibration curve, was calculated to be 0.1694 µA nM^−1^. The LOD and LOQ were calculated according to LOD = 3*σ*/*S* and LOQ = 10*σ*/*S*, respectively, where *σ* represents the standard deviation of five blank measurements and *S* is the slope of the calibration curve. Based on these equations, the LOD and LOQ were determined to be 11.49 nM and 38.29 nM, respectively. These results indicate that the MIP/ASB/GCE sensor can sensitively and reliably quantify PBZ within the examined concentration range.

As summarized in Table 1, the reported PBZ detection methods cover electrochemical, chromatographic, immunological, and optical/MIP-based platforms, with different advantages depending on the target matrix and analytical purpose. The previously reported MIP/B-CuO-Gr/GCE sensor achieved a lower LOD of 3.30 × 10^−10^ M, but relied on a more complex nanocomposite platform. The BDDE based voltammetric method showed a wider working range of 0.5–20 µM, but it did not provide molecularly imprinted recognition. Immunological assays exhibited low detection limits in environmental water, soil, wheat, rice, and fruit/vegetable samples, but their performance is closely associated with antibody-based recognition and matrix-specific assay optimization. Chromatographic methods provided low residue-level quantification in sprout samples, whereas they require more instrument-dependent workflows. In comparison, the present method offers selective recognition, nM-level sensitivity, and successful application in Radix Angelicae Sinensis. These results indicate that the proposed sensor provides a practical alternative for PBZ analysis in a complex herbal matrix while maintaining satisfactory analytical performance.

**Table 1:** Comparison of recent analytical methods for PBZ determination

Category	Method/Platform	Linear range (reported)	LOD/LOQ (reported)	Sample matrix	Ref.
Electrochemical	MIP/B-CuO-Gr/GCE, SWV	1.0 × 10^−9^–1.0 × 10^−8^ M	LOD: 3.30 × 10^−10^ M	Apple juice, orange juice	64
Electrochemical	BDDE-based voltammetric method	0.5–20 µM	LOD: 0.125 µM; LOQ: 2.6 × 10^−7^ M	Pesticide preparations; water	7
Optical/MIP	Magnetic MIP-SERS sensor, FSAA@MIP	0.075–12.75 µg g^−1^	LOD: 0.075 µg g^−1^	Soil	65
Immunological	TRFIA	—	LOD: 0.067 µg L^−1^	Environmental water, soil	13
Immunological	TRFMs-LFIA	—	1.72 ng mL^−1^ (qLOD); 50 ng mL^−1^ (vLOD)	Wheat, rice	15
Immunological	Dual-color ICA	0.5–50 ng mL^−1^ (orange)	LOD: 0.117 µg kg^−1^ (orange)	Orange, grape, cabbage mustard	14
		0.05–50 ng mL^−1^ (grape)	0.109 µg kg^−1^ (grape)		
		0.5–50 ng mL^−1^ (cabbage mustard)	0.131 µg kg^−1^ (cabbage mustard)		
Chromatographic	Mixed SPE column separation + LC-MS/MS	1.0–50 ng mL^−1^	LOD: 0.002 mg kg; LOQ: 0.005 mg kg^−1^	Soybean sprouts; mung bean sprouts	66
This work	MIP/ASB/GCE, DPV	50–450 nM	LOD: 11.49 nM; LOQ: 38.29 nM	Radix Angelicae Sinensis	This study

### 3.6 Selectivity of the sensor

The selectivity of the proposed sensor was evaluated using triazophos, ferulic acid, and glyphosate as potential interfering substances, each tested at the same concentration as PBZ (250 nM). As shown in Fig. 8c and S5, the MIP electrode exhibited a significantly stronger response toward PBZ than toward the three interferents, whereas the NIP electrode showed only weak responses to all tested species. This outcome suggests that the prominent signal observed for PBZ mostly derives from the distinct recognition cavities formed during the imprinting process, rather than from generic adsorption into the polymer matrix. Despite the three interferents eliciting minor responses on the MIP electrode, their Δ*I* values were significantly lower than that of PBZ, so affirming that the imprinted interface demonstrates adequate selectivity for the target molecule. The concurrent incorporation of structurally distinct pesticides and a representative endogenous component from the Angelica matrix reinforces the anti-interference efficacy of the proposed sensing platform.

### 3.7 Reproducibility, repeatability, and stability

The reproducibility of the fabricated sensor was assessed using six independently prepared MIP electrodes under the optimized conditions. After template removal, all electrodes were incubated with 250 nM PBZ, and the corresponding Δ*I* values were recorded. As shown in Fig. 8d, the six electrodes produced relatively consistent responses, with RSD of 5.49%, indicating acceptable fabrication reproducibility. This result suggests that the preparation procedure of the ASB-supported MIP interface is sufficiently reliable to yield comparable sensing performance from electrode to electrode.

To further evaluate the repeatability of the sensor, a single MIP electrode underwent continuous adsorption and elution cycles. The sensor demonstrated reliable performance over five cycles, with RSD of 6.30%, thus confirming its repeatability.

The long-term storage stability of the fabricated sensor was investigated under dry refrigerated conditions. After template elution, the electrodes were thoroughly dried and stored at 4 °C in a sealed container with silica gel. As shown in Fig. 8e, the sensor retained 93.06%, 86.63%, and 82.90% of its initial response after 7, 14, and 21 days, respectively. The gradual decrease in response may be attributed to slight changes in the imprinted polymer layer and interfacial charge-transfer properties during storage. Nevertheless, more than 80% of the original response was maintained after 21 days, indicating that the ASB/MIP/GCE sensor possesses acceptable storage stability for practical PBZ detection.

### 3.8 Real sample analysis

To evaluate the practical applicability of the proposed sensor, recovery experiments were carried out in Radix Angelicae Sinensis extracts using the standard addition method. Representative DPV curves of the sensor after incubation in unspiked and PBZ-spiked real sample extracts are shown in Fig. S6. With increasing spiked PBZ concentration, the peak current of the Fe(CN)_6_^3−/4−^ redox probe gradually decreased, indicating enhanced PBZ rebinding within the imprinted cavities and stronger blocking of probe transport.

As shown in Table 2, after spiking the real sample at three concentration levels (80, 250, and 400 nM), the obtained recoveries ranged from 105.80% to 109.30%, with RSD values of 3.10–4.84% (*n* = 3).

**Table 2:** Recoveries of the constructed MIP sensor in real samples

Real sample	Added (nM)	Found by the proposed sensor (nM)	Recovery (%)	RSD(%, *n* = 3)
Radix Angelica Sinensis	80	84.64	105.80	4.84
	250	270.92	108.37	3.10
	400	437.20	109.30	3.99

These results indicate that the developed MIP/ASB/GCE sensor can provide accurate and reasonably precise determination of PBZ in the Angelica matrix, despite the complexity of the herbal sample background. The slightly higher-than-100% recoveries suggest that matrix effects may contribute to a modest signal enhancement, but the values remain within an acceptable analytical range for spiked recovery analysis. Overall, the satisfactory recovery and low RSD values demonstrate that the proposed sensor possesses good practical applicability for PBZ analysis in real traditional Chinese medicine samples.

## 4 Conclusion

In this study, a novel molecularly imprinted electrochemical sensor was successfully constructed for the selective and sensitive determination of PBZ in Radix Angelicae Sinensis. The sensor design was built upon a sustainable platform: porous biochar derived from discarded Angelica sinensis stalks served as an eco-friendly and highly conductive substrate, while a molecularly imprinted polymer layer was rationally developed using a combined computational approach. DFT calculations enabled the systematic screening of *o*-phenylenediamine as the optimal functional monomer, and Forcite molecular dynamics simulations further determined the ideal template-to-monomer ratio, ensuring the formation of high-affinity recognition cavities. In addition, DES-assisted elution was employed to remove the template while preserving the accessibility of imprinted cavities, thereby improving the rebinding response of the MIP sensor in a greener manner.

The optimized MIP/ASB/GCE sensor exhibited a linear response from 50 to 450 nM, with an LOD of 11.49 nM and an LOQ of 38.30 nM. It showed acceptable selectivity, reproducibility, repeatability, and three-week storage stability. More importantly, the practical applicability of the sensor was validated through successful recovery tests in spiked Radix Angelicae Sinensis samples, highlighting its potential as a practical tool for quality control of herbal medicines.

Beyond providing a reliable method for PBZ monitoring in complex herbal matrices, this work establishes a comprehensive computational–experimental platform for the rational design of high-performance molecularly imprinted sensors. The integration of DFT-guided monomer selection, molecular dynamics-based stoichiometric optimization, and green synthesis strategies represents a generalizable framework that can be extended to the detection of other pesticide residues or analytes in challenging real-world samples.

## Author contributions

Xin Wang: conceptualization, methodology, writing – original draft. Xuxia Liu: data curation. Pen Jin: resources, data curation. Delai Zhou: methodology. Guodi Lu: writing – original draft. Jia Hou: supervision, funding acquisition. Shijun Shao: writing – review & editing. Jian Xu: Materials Studio calculation, writing – original draft. Fude Yang: conceptualization, writing – review & editing, supervision, funding acquisition.

## Conflicts of interest

The authors declare that they have no known competing financial interests or personal relationships that could have appeared to influence the work reported in this paper.

## Supplementary Material

RA-016-D6RA02926D-s001

## Data Availability

Data will be made available on request. The data supporting this article are included in the main text and the supplementary information (SI). Supplementary information: additional DFT calculations, textural and morphological characterization of ASB, EDS elemental composition, fitted EIS parameters, electroactive surface-area estimation, and supporting DPV data for eluent optimization, selectivity, and real-sample analysis. See DOI: https://doi.org/10.1039/d6ra02926d.
