# Supplementary material for: Computationally optimized molecularly imprinted electrochemical sensor based on biomass-derived biochar for paclobutrazol analysis in Radix Angelicae Sinensis
Source: RSC Adv. 2026 Jul 3;16(35):35249–62. doi: 10.1039/d6ra02926d (PMC13329671; doi:10.1039/d6ra02926d)
Supplement: RA-016-D6RA02926D-s001 [file RA-016-D6RA02926D-s001.pdf]

Supplementary Information (SI) for RSC Advances.
This journal is © The Royal Society of Chemistry 2026

# Supplementary Information

# Computationally Optimized Molecularly Imprinted Electrochemical Sensor Based on Biomass-derived biochar for Paclobutrazol Analysis in Radix Angelicae Sinensis

Xin Wang [a,b], Xuxia Liu[a],Pen Jin[b], Delai Zhou [a], Guodi Lu[a], Jia Hou [a], Shijun Shao [c] ,Jian Xu [c]and Fude Yang [a,*]

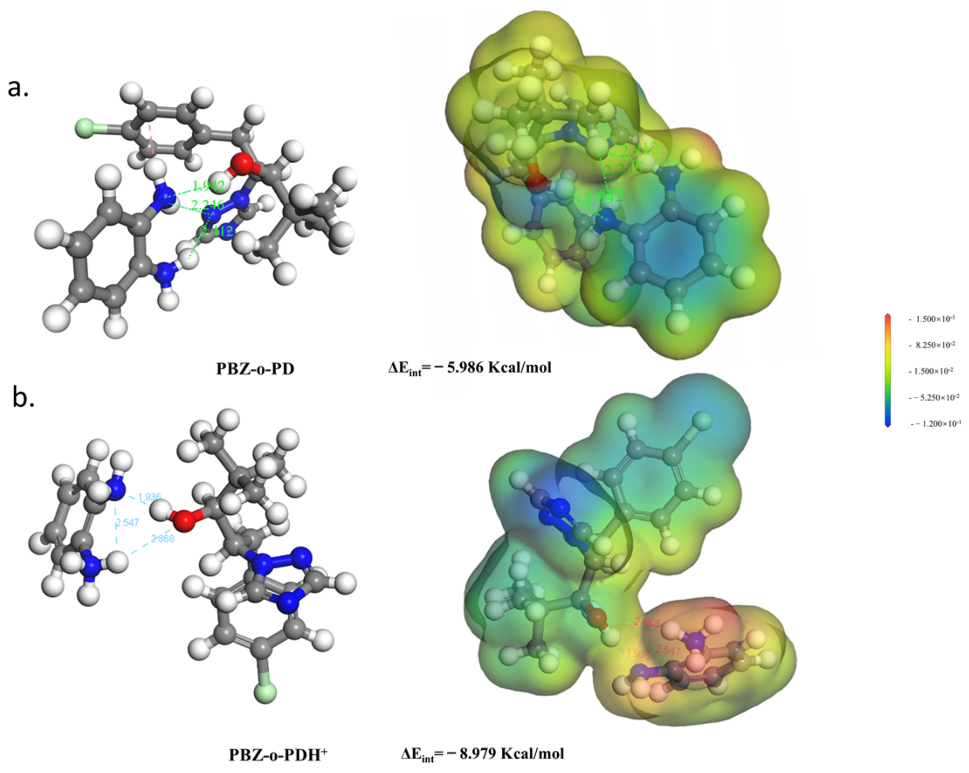


**Figure S1.** DFT-optimized interaction models of PBZ with neutral and monoprotonated o-PD.

**Table S1.** Textural parameters of ASB derived from $N_2$ adsorption-desorption analysis

| Sample | BET surface area ($m^2\ g^{-1}$) | Micropore area ($m^2\ g^{-1}$) | Total pore volume ($cm^3\ g^{-1}$) | Micropore volume ($cm^3\ g^{-1}$) | Average pore diameter (nm) |
|---|---|---|---|---|---|
| ABS | 896.61 | 887.14 | 0.3674 | 0.3475 | 1.64 |

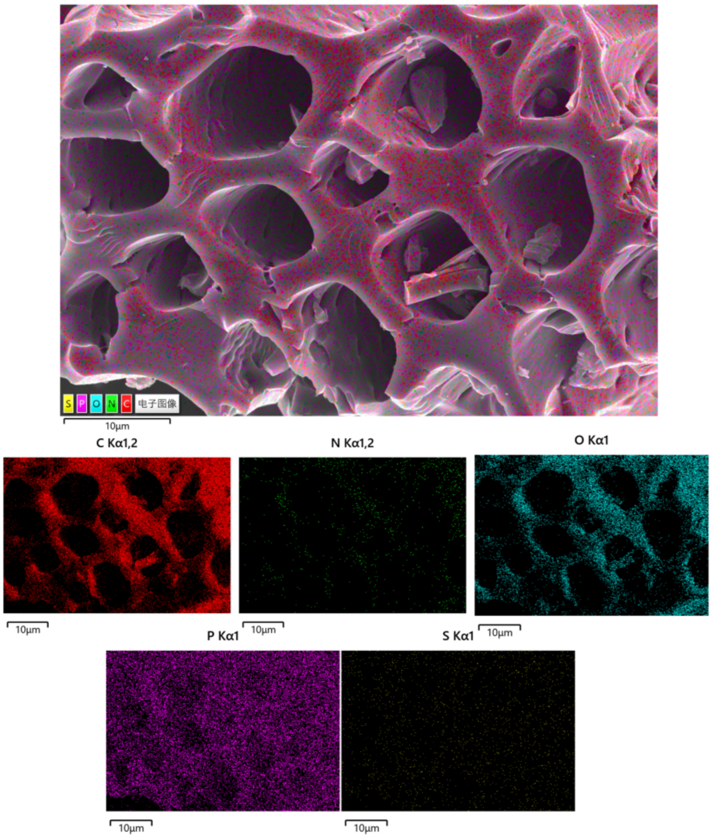


**Figure S2.** SEM images of ASB and corresponding elemental mapping images.

**Table S2** Elemental composition of the ASB obtained from EDS results.

| Sample | C (at,%) | N (at,%) | O (at,%) | P (at,%) | S (at,%) |
|---|---|---|---|---|---|
| ABS | 79.33 | 0.76 | 14.37 | 5.43 | 0.10 |

**Table S3.** Fitted electrochemical impedance parameters of different electrodes based on the equivalent circuit Rs–(Rp || CPE)–Ws

| Electrode | Rs / Ω | Rp / Ω | CPE-T | CPE-P | Ws-R | Ws-T | Ws-P | $\chi^2$ |
|---|---|---|---|---|---|---|---|---|
| **Bare GCE** | 89.1 | 121.2 | $6.33 \times 10^{-6}$ | 0.766 | 6563 | 138.6 | 0.395 | $9.68 \times 10^{-4}$ |
| **ASB/GCE** | 94.1 | 21.8 | $2.12 \times 10^{-5}$ | 0.654 | 5701 | 98.2 | 0.468 | $8.82 \times 10^{-4}$ |
| **MIP/ASB/GCE (before elution)** | 87.6 | 1727 | $1.65 \times 10^{-6}$ | 0.872 | 8168 | 58.9 | 0.407 | $1.23 \times 10^{-3}$ |
| **MIP/ASB/GCE (after elution)** | 86.9 | 893.8 | $1.58 \times 10^{-6}$ | 0.916 | 9674 | 251.7 | 0.352 | $4.36 \times 10^{-4}$ |

**Note:** Rs represents the solution resistance, Rp corresponds to the interfacial charge-transfer resistance, CPE denotes the constant phase element, and Ws represents the finite-length Warburg diffusion element.

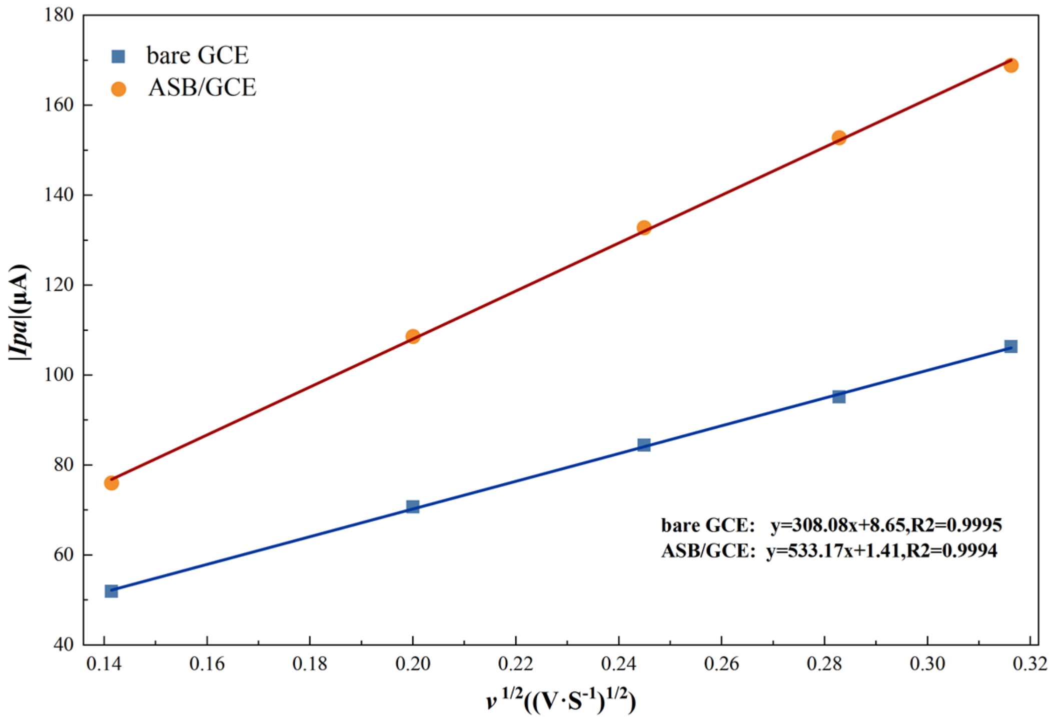


**Figure S3.** Electroactive surface area estimation of bare GCE and ASB/GCE

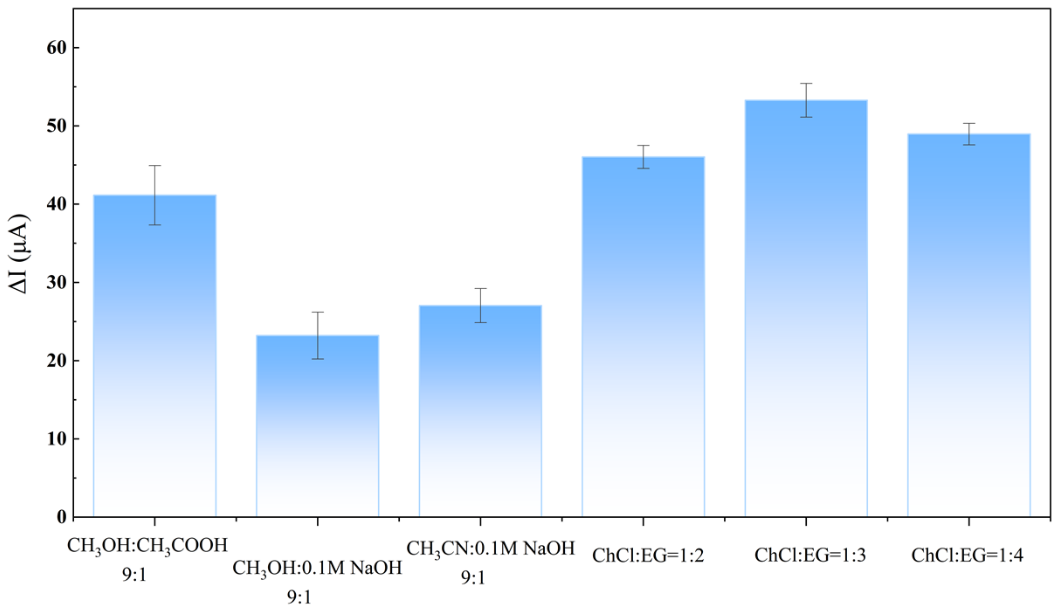


**Figure S4.** Comparison of DPV-derived current differences after PBZ rebinding using different eluents.

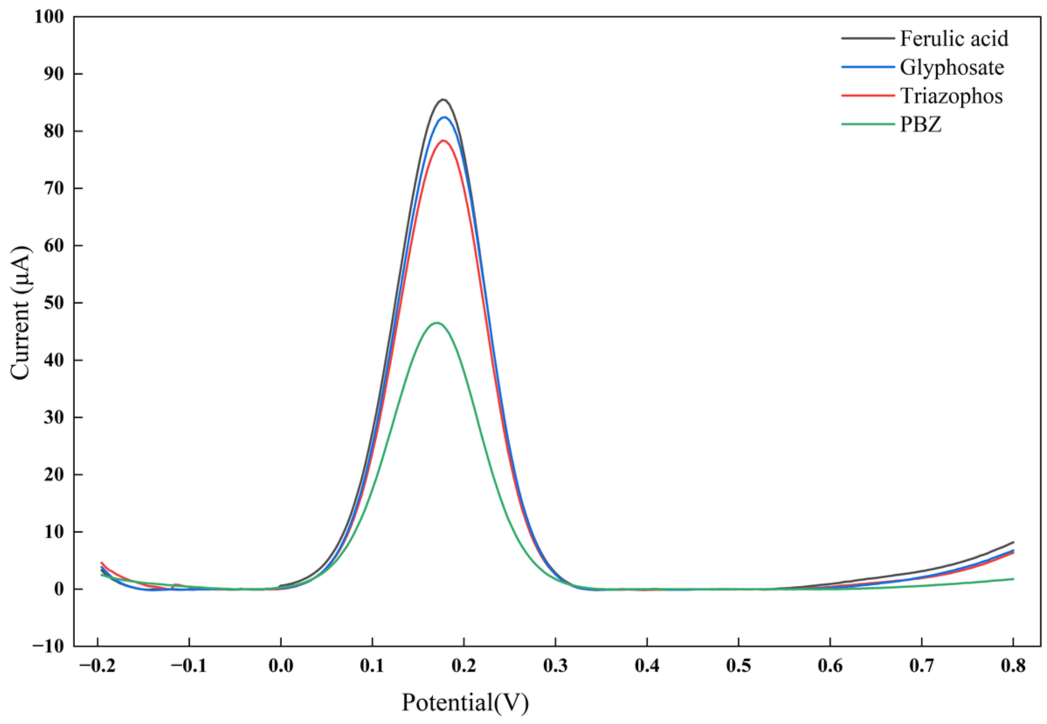


**Figure S5.** DPV responses of the MIP/ASB/GCE sensor after incubation with PBZ and interfering compounds.

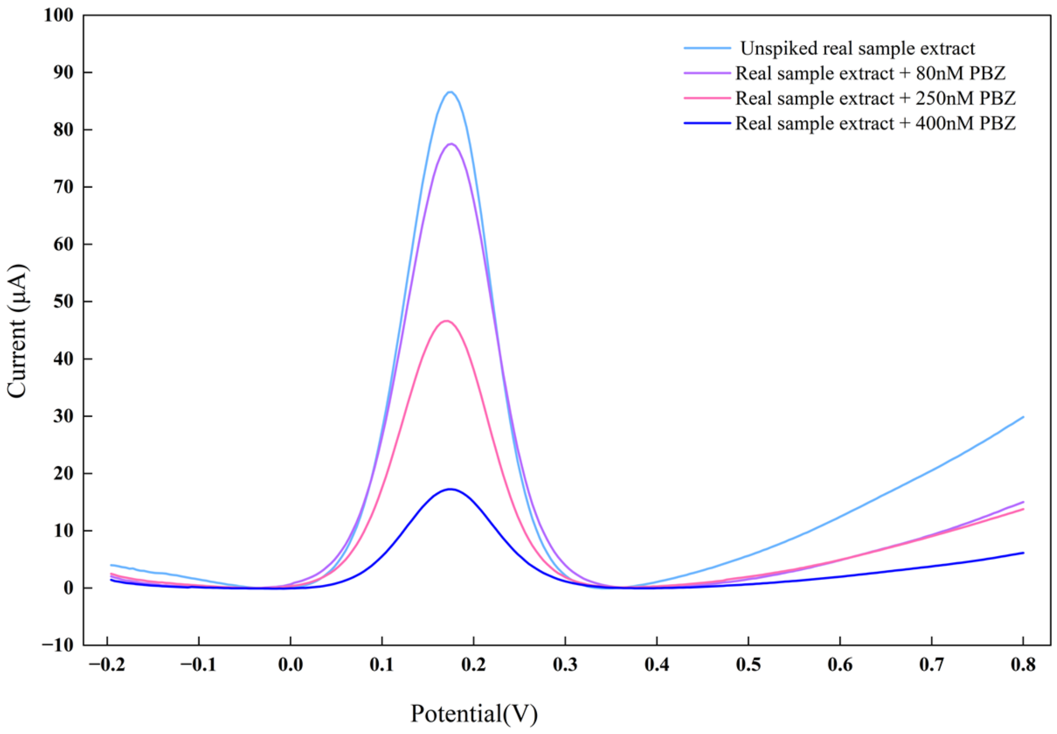


**Figure S6.** Representative DPV curves of the MIP/ASB/GCE sensor after incubation in Radix Angelicae Sinensis extracts spiked with different PBZ concentrations. The curves correspond to the unspiked real sample extract and extracts spiked with 80, 250, and 400 nM PBZ.
